# Comparative survival of cancer patients requiring Israeli permits to exit the Gaza Strip for health care: A retrospective cohort study from 2008 to 2017

**DOI:** 10.1371/journal.pone.0251058

**Published:** 2021-06-02

**Authors:** Benjamin Bouquet, Francesco Barone-Adesi, Mohamed Lafi, Kathryn Quanstrom, Federica Riccardi, Henry Doctor, Walaa Shehada, Juliana Nassar, Sali Issawi, Mahmoud Daher, Gerald Rockenschaub, Arash Rashidian

**Affiliations:** 1 WHO Occupied Palestinian Territory, Jerusalem, Israel; 2 Department of Translational Medicine and CRIMEDIM-Research Center in Emergency and Disaster Medicine, University of Eastern Piedmont, Novara, Italy; 3 University of Michigan Medical School, Ann Arbor, MI, United States of America; 4 Independent Researcher, Hautes-Alpes, L’Argentière la Bessée, France; 5 Department of Science, Information and Dissemination, WHO Regional Office for the Eastern Mediterranean, Nasr City, Cairo, Egypt; Chang Gung Memorial Hospital and Chang Gung University, Taoyuan, TAIWAN

## Abstract

**Background:**

Gaza has been under land, sea, and aerial closure for 13 years, during which time Palestinian patients from Gaza have been required to obtain Israeli-issued permits to access health facilities in the West Bank (including east Jerusalem), as well as in Israel and Jordan. Specific groups, like cancer patients, have a high need for permits due to lack of services in Gaza. The approval rate for patient permits to exit Gaza dropped from 94% in 2012 to 54% in 2017. We aimed to assess the impact of access restrictions due to permit denials/delays on all-cause mortality for cancer patients from Gaza referred for chemotherapy and/or radiotherapy.

**Methods:**

This study matched 17,072 permit applications for 3,816 cancer patients referred for chemotherapy and/or radiotherapy from 1 January 2008 to 31 December 2017 with referrals data for the same period and mortality data from 1 January 2008 to 30 June 2018. We carried out separate analyses by period of first application (2008–14; 2015–17), in light of varying access to Egypt during these times. Primary analysis compared survival of patients according to their first referral decision (approved versus denied/delayed) using Kaplan-Meier method and Cox regression.

**Findings:**

Mortality in patients unsuccessful in permit applications from 2015–17 was significantly higher than mortality among successful patients, with a hazard ratio of 1·45 (95% CI: 1·19–1·78, p<0.001), after adjusting for age, sex, type of procedure, and type of cancer. There was no significant difference in mortality risk for the two groups in the 2008–2014 period.

**Interpretation:**

Limitations to patient access due to unsuccessful applications for permits to exit the Gaza Strip had a significant impact on mortality for cancer patients applying for chemotherapy and/or radiotherapy in the period 2015–17. The substantially higher number of annual unsuccessful permit applications from 2015, combined with severely limited alternatives to access chemotherapy and radiotherapy during these years, may be important factors to explain the difference in the impact of permits delays/denials between the two study periods.

## Introduction

The Palestinian territory occupied by Israel since 1967 (oPt) comprises the West Bank including east Jerusalem and the Gaza Strip, demarcated by the 1949 Armistice lines [1]. The ability of Palestinians to move between parts of the oPt and outside of it has varied considerably since the start of Israel’s occupation [2, 3]. The first fence around the Gaza Strip was erected in 1994 [4], with subsequent rebuilding and reinforcement. This and the 2005 Israeli withdrawal of its settlers [5] enabled the closure and blockade of Gaza by land, sea, and air from 2007 [6]. Closure has left just two entry/exit points, at Beit Hanoun/Erez in the north and Rafah in the south [1], with movement for Palestinians across Beit Hanoun/Erez in the north dependent on obtaining an Israeli-issued permit.

By 2014, over 100 types of Israeli-issued permits existed for Palestinians–of which permits issued to patients and companions to travel for health reasons comprise one category [7]. The Ministry of Health (MoH) of the Palestinian Authority, established following the Oslo Accords in the early 1990s, administers the public health care system for Palestinians in the oPt [8] and depends on the procurement of referral services for provision of essential health care that is unavailable in Palestinian public hospitals. Israeli permits are required for all referrals made from the Gaza Strip to health facilities in the West Bank, including east Jerusalem, Israel [9], and Jordan [10]. Similarly, most referrals from the West Bank require Israeli-issued permits to reach east Jerusalem, Israel, or Jordan [11].

From 2008 to 2017, there were 153,037 permit applications to Israeli authorities for the exit of Palestinian patients from the Gaza Strip [20]. There has been a dramatic reduction in the approval rate over time, declining from a high of 94% in 2012 to 54% in 2017 [12]. Excluding permit applications recorded as having two conflicting responses (158) or no response (61), from 2008 to 2017, 74% (112,544) of patient permit applications were approved; 23% (34,608) were categorized by WHO as ‘delayed’, meaning that patients received no definitive response to their permit applications by the date of their hospital appointments; while 4% (5,666) were denied, meaning that those patients received notification from Israeli authorities that their permits had been denied [13].

**Fig 1:**
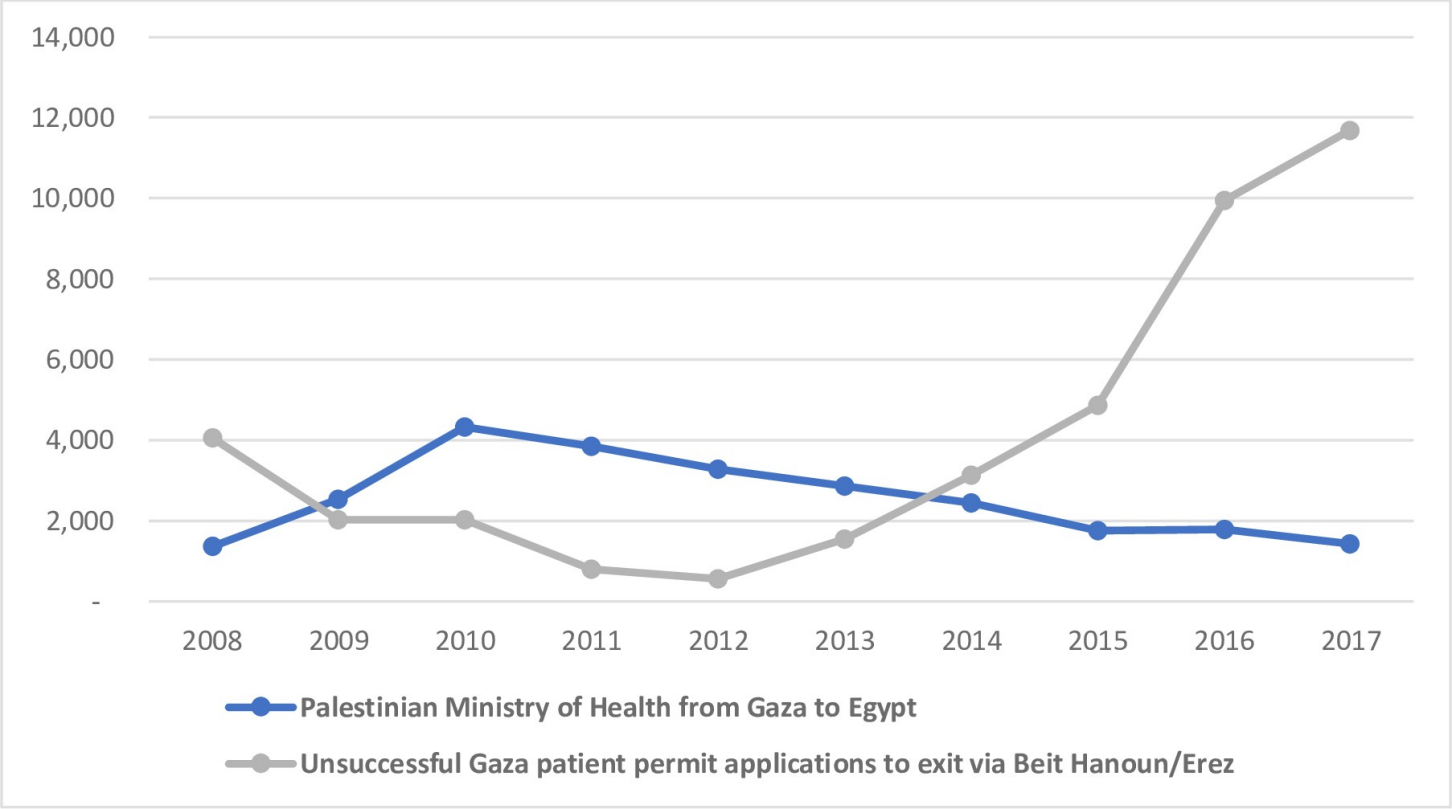
Annual number of referrals issued by the Palestinian Ministry of Health from Gaza to Egypt, compared to annual number of unsuccessful permit applications by Gaza patients to Israeli authorities [13, 15].

Access through Rafah crossing to Egypt was the only other possible exit point for Palestinians in the Gaza Strip during the years of this study, presenting a potential alternative route to accessing health care for referral patients, without requiring Israeli-issued permits to travel. However, the possibility of movement across Rafah varied considerably from 2008 to 2017, reflected in the annual numbers of person-crossings. The recorded annual number of exits of Palestinians from Gaza to Egypt increased from 11,079 in 2008 to peak at 208,376 in 2012. After this, there was a substantial decline, to 14,292 in 2015, with annual numbers remaining relatively low thereafter [14]. The annual number of MoH referrals to Egypt increased from 2008 to peak in 2010 at 4,328, declining prior to the peak in the overall annual crossings through Rafah in 2012 [15]. In this year (2012), the approval rate for Israeli permits for patients to exit via Beit Hanoun/Erez was at its highest (94%) [13]. From 2015 to 2017, the number of referrals to Egypt was consistently and substantially lower than the number of unsuccessful Israeli permit applications to exit via Beit Hanoun/Erez in the north (see Fig 1) [13, 15], indicating that the possibility of alternative routes to treatment for referral patients during this period was severely constrained.

Cancer patients have a particularly high need for referral out of the Gaza Strip, with the major Palestinian oncology centre at Augusta Victoria Hospital in east Jerusalem and a shortage of specialists, lack of radiotherapy and nuclear medicine facilities, and chronic shortages in essential cancer chemotherapy medicines in Gaza [9]. 8,927 of the 44,812 (20%) patients applying for permits between 2008 and 2017 had a diagnosis of cancer [13]. Permit applications for this group of patients accounted for 56,479 of all 153,037 (37%) patient permit applications during the period [13]. Clinically, the likelihood of an impact of barriers to accessing essential chemotherapy medicines and radiotherapy for cancer patients on mortality is high. For this reason, cancer patients applying for chemotherapy and/or radiotherapy were the focus of this research.

Universal health coverage envisages equitable and non-discriminatory access to healthcare and is enshrined in WHO mandates and resolutions as well as being one of the core minimum obligations placed on States for fulfilment of the right to the highest attainable standard of physical and mental health [16–18], which is guaranteed under international human rights law applicable to the occupied Palestinian territory [19–21]. For Palestinians in the oPt, the right to health is further guaranteed by customary international law and international humanitarian law including the laws of occupation [22–25].

The objective of this study is to assess the health impact of unsuccessful permit applications (denials/delays) on all-cause mortality for cancer patients in Gaza referred for chemotherapy and/or radiotherapy between 2008 and 2017. The study hypothesized that unsuccessful permit applications would be associated with increased mortality, where alternative access to health care was not otherwise possible.

## Materials and methods

### Study design

We conducted a retrospective cohort study of cancer patients making permit applications to exit the Gaza Strip for chemotherapy and/or radiotherapy from 1 January 2008 to 31 December 2017. Patients whose initial permit application was delayed or denied (exposed group) were compared with those whose initial permit application was accepted (reference group). The primary outcome measure assessed in the study was survival of patients from date of first permit application.

We collected mortality data for all patients, matching permit applications with mortality data from the Gaza death registry. Mortality data was collected for the period of 1 January 2008 to 30 June 2018, provided by the MoH. Permits data from the Gaza Health Liaison Office (HLO) included information on patient age, sex, permit application outcome, diagnosis by ICD-10 coding description, date of application, date of appointment, urgency of appointment, referral specialism, reason for referral, referral destination, and referral hospital. Because patients applying for Israeli permits require medical and financial approval of their referrals from the MoH, we also matched permit applications data from HLO with data collected on referral needs for the same patients from the MoH, using encrypted personal identification numbers. This provided additional information on reason for referral and referral specialism.

The Helsinki Committee for Ethical Approval of the Palestinian Health Research Council provided written approval for carrying out this research (approval number PHRC/HC/517/19). The study analysed anonymized data, giving full consideration to the storage, sharing and communication of potentially sensitive information.

### Statistical analysis

Unadjusted survival analysis was carried out using the Kaplan-Meier method. Multivariable Cox regression models were constructed to compare mortality hazards for any cause between patients who had their initial application approved and those who had their initial application delayed or denied. Time since the first application for either chemotherapy or radiotherapy was chosen as the start time in the survival analysis. Patients were censored at death or at the end of follow up (30 June 2018), whichever occurred first. Cox models were adjusted for the following covariates: age, sex, type of cancer, type of treatment (chemotherapy, radiotherapy, or both), and urgency of the referral. Type of cancer was included in the model as a random effect (75 groups based on the first three digits of the ICD-10 code). We carried out separate analyses by period of first application (2008–14 and 2015–17), in light of varying access across Rafah during these two periods. The assumption of proportional hazards was evaluated by means of graphical checks on the log cumulative hazard for each covariate and a formal test based on Schoenfeld residuals.

Several secondary analyses were conducted to further evaluate the association between success of the first application and survival. First, we analysed separately patients referred for chemotherapy and radiotherapy. Second, we reran the analysis and included only non-urgent referrals, which were expected to be the most affected by the decline in approval rate. Third, we analysed separately patients who were always unsuccessful with their applications and those who had their application eventually approved. Finally, we explored the possible interaction between application time and permit approval. The functional form of the interaction was thoroughly evaluated using Subpopulation Treatment-Effect Pattern Plots (STEPP), as suggested by Royston and Sauerbrei [26].

Results of the Cox analysis were expressed in terms of estimated hazard ratios and 95% confidence intervals. All statistical tests were two-tailed. P-values below 0.05 were considered statistically significant. Analyses were performed with the software Stata 12 (StataCorp, College Station, TX, USA).

## Results

During the study period, 3,816 cancer patients referred for chemotherapy and/or radiotherapy made 17,072 permit applications to exit the Gaza Strip to obtain chemotherapy and/or radiotherapy. 3,161 patients (83% of the total) had their initial permit application approved, while 655 patients had their initial application delayed or denied. Of these, 386 patients (59%) had a later permit application approved and were analysed separately in the secondary analysis. For these patients, the mean delay time from their initial application to their first successful hospital appointment was 77 days (SD 162).

The most common cancer diagnoses for patients applying for permits to exit Gaza for chemotherapy and/or radiotherapy were breast cancer, lymphoid and hematopoietic malignancies, and gastro-intestinal cancer, accounting for about 60% of all cancers (Table 1). The proportion of these neoplasms was stable between the two groups and in the two study periods. The groups used in primary analysis were also comparable in terms of the percentage of referrals that were non-urgent (94% in the first period and 88% in the second period). Patients with initial applications approved had a larger number of total applications per patient, a higher average age at application, and were more likely to be female, compared to the group initially not approved (Table 1). Overall data quality and comprehensiveness was high for the datasets utilized in the study. S1 Table provides a list of indicators used to examine data quality and comprehensiveness, including missing data on sex and diagnosis, and occurrence of death before permit application.

**Table 1:** Baseline characteristics of cohorts of cancer patients referred for chemotherapy and/or radiotherapy in the periods 2008–2014 and 2015–2017.

	Period 2008–2014		Period 2015–2017	
	Initial permit application not approved (N = 238)	Initial permit application approved (N = 2008)	Initial permit application not approved (N = 417)	Initial permit application approved (N = 1153)
Females	117 (49%)	1222 (61%)	234 (56%)	681 (59%)
Mean (SD) age at application	43.0 (18.9)	48.7 (19.0)	46.2 (16.0)	48.8 (20.2)
Mean (SD) follow up (years)	4.60 (3.71)	3.68 (3.07)	1.18 (0.76)	1.66 (0.99)
Mean (SD) number of applications per patient	3.72 (3.97)	4.16 (6.21)	4.34 (4.30)	5.22 (6.11)
Type of cancer				
Breast	56 (24%)	634 (32%)	144 (35%)	320 (28%)
Lymphoid and hematopoietic	42 (18%)	297 (15%)	66 (16%)	231 (20%)
Gastro-intestinal	27 (11%)	267 (13%)	54 (13%)	177 (15%)
Other	113 (47%)	810 (40%)	153 (36%)	425 (37%)
Type of treatment				
Initially referred for chemotherapy	83 (35%)	720 (36%)	157 (38%)	550 (48%)
Initially referred for radiotherapy	137 (57%)	1134 (56%)	216 (51%)	496 (43%)
Initially referred for both chemotherapy and radiotherapy	18 (8%)	154 (8%)	44 (11%)	107 (9%)
Type of referral				
Non-urgent	225 (94%)	1883 (94%)	366 (88%)	1021 (88%)
Urgent or life-saving	13 (6%)	125 (6%)	51 (12%)	132 (12%)

Values are frequencies (percentages) of participants unless stated otherwise.

### Comparative survival analysis

**Fig 2:**
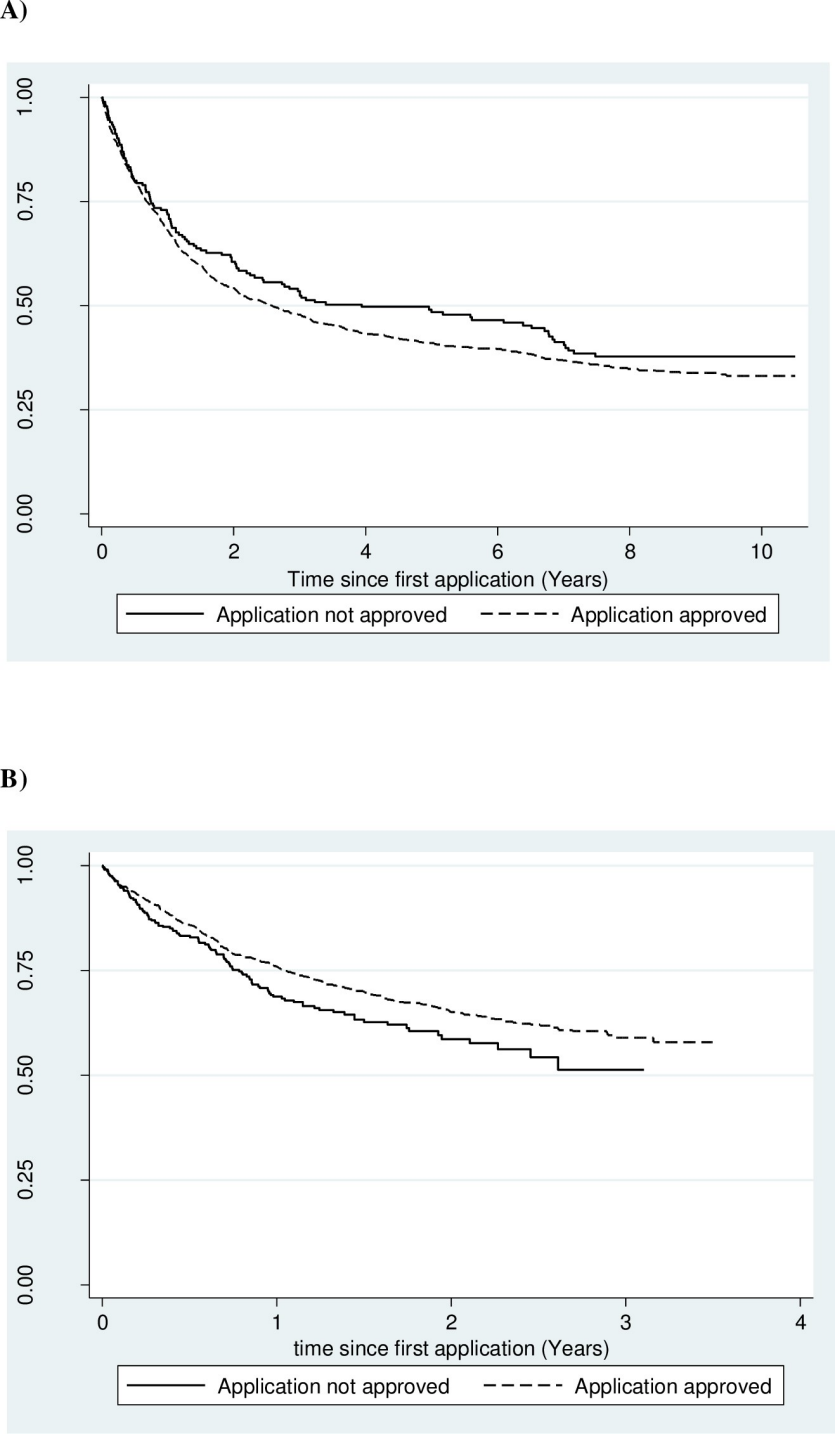
Kaplan-Meier survival estimates of cancer patients, stratified by approval of their first permit application for chemotherapy or radiotherapy. Results for period 2008–2014 (A) and period 2015–2017 (B).

There were 1,830 deaths (48%) among 3,816 individuals over the study period. The unadjusted survival of the two groups is reported in Fig 2, while results from multivariable Cox regression are reported in Table 2. From 2008 to 2014, the risk of death for patients who were unsuccessful in their first permit applications was comparable with that of successful patients (Hazard Ratio [HR] 0·84; 95% Confidence Interval [CI] 0·69–1·01; p-value: 0·07). However, in the period 2015–2017 the risk of death for patients whose first application was not approved was substantially higher than that of successful patients (HR 1·45; 95% CI 1·19–1·78; p-value: <0.001) (Table 2).

**Table 2:** Risk of death for cancer patients unsuccessful in applying for permit applications, compared to those successful.

		Initial permit application approved		Initial permit application not approved		
Period		Cases/person-years	HR (95% CI)	Cases/person-years	HR (95% CI)	P value
**2008–2014**	Crude	1188/7406	1 (ref)	126/1095	0.81 (0.68–0.98)	0.03
	Adjusted	1188/7406	1 (ref)	126/1095	0.84 (0.69–1.01)	0.07
**2015–2017**	Crude	375/1923	1 (ref)	141/ 493	1.29 (1.06–1.57)	0.01
	Adjusted	375/1923	1 (ref)	141/ 493	1.45 (1.19–1.78)	<0.001

Results displayed by period of first application.

*Results adjusted for sex, age, type of treatment, type of cancer and urgency of the referral

**Fig 3:**
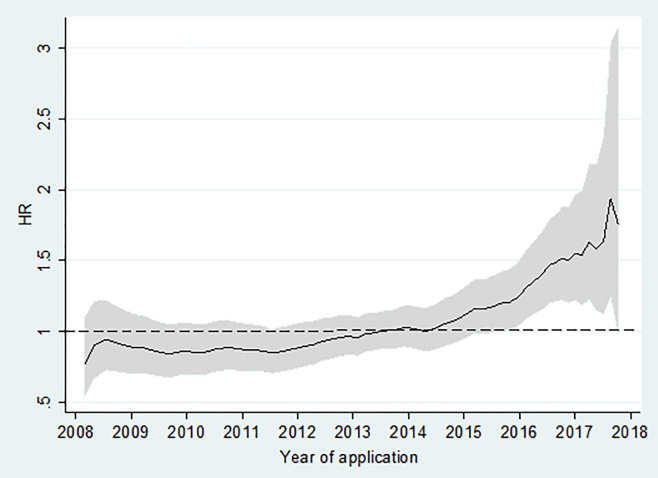
Risk of death for cancer patients unsuccessful in applying for permit applications compared to those successful, by year of first application.

Results did not substantially change in the secondary analyses that we carried out. The effect remained similar to those of the main analysis when we analysed separately patients referred for chemotherapy and radiotherapy, and when we included only non-urgent referrals in the analysis (Table 3). We also found similar results when we analysed separately patients who were always unsuccessful with their applications and those who had their application eventually approved. Both groups displayed a similar risk of death to approved patients in the first period and an increased risk in the second period (Table 4). There was a clear interaction between application time and approval status (LR test, p value = 0.0007). Fig 3 shows how the effect of approval on mortality changed over time. While the HR was around 1 until 2014, there was a clear increase afterwards.

**Table 3:** Risk of death for cancer patients unsuccessful in applying for permit applications, compared to those successful.

		Initial permit application approved		Initial permit application not approved	
Period		Cases/person-years	HR (95% CI)	Cases/person-years	HR (95% CI)
**2008–2014**	Chemotherapy only*	448/ 2418	1 (ref)	47/355	0.85 (0.62–1.16)
	Radiotherapy only*	654/4433	1 (ref)	68/658	0.78 (0.60–1.02)
	Non-urgent referrals only*	1104/7044	1 (ref)	118/ 1040	0.85 (0.70–1.03)
**2015–2017**	Chemotherapy only*	201/855	1 (ref)	61/165	1.45 (1.08–1.95)
	Radiotherapy only*	133/892	1 (ref)	66/269	1.59 (1.15–2.20)
	Non-urgent referrals only*	316/1761	1 (ref)	127/435	1.69 (1.36–2.09)

Secondary analyses. Results displayed by period of first application.

*Results adjusted (where appropriate) for sex, age, type of treatment, type of cancer and urgency of the referral

**Table 4:** Risk of death for patients for patients initially not approved and later approved and patients always not approved, compared to patients whose initial permit applications was approved.

		Initial permit application approved		Initial permit application not approved, subsequently approved		Permit application never approved	
Period		Cases/person-years	HR (95% CI)	Cases/person-years	HR (95% CI)	Cases/person-years	HR (95% CI)
**2008–2014**	Crude	1188/ 7406	1 (ref)	78/677	0.80 (0.63–1.00)	48/418	0.85 (0.64–1.13)
	Adjusted	1188/ 7406	1 (ref)	78/677	0.85 (0.67–1.07)	48/418	0.82 (0.61–1.10)
**2015–2017**	Crude	375/ 1923	1 (ref)	93/331	1.31 (1.04–1.64)	48/162	1.26 (0.93–1.71)
	Adjusted	375/ 1923	1 (ref)	93/331	1.48 (1.17–1.87)	48/162	1.41 (1.03–1.91)

Results displayed by period of first application.

*Results adjusted for sex, age, type of treatment, type of cancer and urgency of the referral

## Discussion

This is the first population-based study to evaluate the health impact of permits delays and denials in Gaza. Our results show a significant association between delay or denial of permit application to Israeli authorities and increased mortality for cancer patients referred for chemotherapy and/or radiotherapy during the period 2015–2017. The risk of death for patients unsuccessful in their initial permit applications during this period was 1·45 times that of patients who were successful. The association between delay or denial of permit applications and increased mortality for this period remained when analysis was restricted to non-urgent applications. Similarly, the association persisted when analysis for the unsuccessful group was separated for patients who were always unsuccessful in their applications and those who had their application eventually approved. The latter finding points to the importance of timely access to cancer treatment for these patients.

During the period 2008–2014 there was no difference in the survival of patients whose initial application was delayed/denied compared to those approved, both in primary and secondary analyses. The annual number of unsuccessful permit applications during these years was substantially lower than for the period 2015–17, which witnessed a sharp increase in numbers not approved. This, and the relative lack of potential alternative routes to accessing care–particularly through Rafah terminal to Egypt–may be plausible explanations for the different apparent effect of permit delays or denials from 2008 to 2014. Availability of essential chemotherapy medicines may also have constituted an alternative route to access care for chemotherapy referrals, however the specific type of chemotherapy needed was not listed in referrals data to support or refute this hypothesis through comparison with data on essential medicines shortages, which was only available disaggregated for different classes of drugs from 2013.

The strengths of this study include the large dataset and reliable data sources, with comprehensive systems for data collection and few internal inconsistencies when examining data quality (S1 Table). It was possible to follow patients for a relatively long period, enough to show the effect on survival of limited access to chemotherapy and radiotherapy. Moreover, the presence of a population registry in Gaza, and strict controls on population movement, reduced potential loss to follow up.

In terms of limitations, mortality is a narrow outcome measure. Lack of availability of essential medicines and the lengthy and unpredictable process of applying for permits to access health care also have implications for patient morbidity, including pain control and mental health. There was a lack of information about socioeconomic status, functional capacity, and comorbidities of the patients. We note, however, that these possible confounders are unlikely to have changed between the two study periods (2008 to 2014; 2015 to 2017) to adequately explain the different results observed in these two periods, or the change in risk of death over time (see Fig 3). Similarly, lack of information on specific cause of death–with assessment instead of all-cause mortality–would be expected to dilute any significant results, due to non-differential misclassification. There was also lack of information on whether the treatment reason for referral (chemotherapy and/or radiotherapy) was actually delivered. Again, non-delivery of treatment to patients exiting Gaza would dilute the effect size–just as subsequent access to treatment within Gaza for unsuccessful permit applicants, for example through drug delivery or the use of the referral system as a means of rationing existing stocks, would lead to cross-over of exposed and non-exposed groups.

For patients in the Gaza Strip diagnosed with breast cancer and colon cancer from 2005 to 2014, Panato et al. found they had respective 5-year survival rates of 65·1% and 50·2%. These survival rates are comparable to surrounding Arab countries during the same period, but substantially lower than high income Mediterranean countries, such as Italy or among Jewish patients in Israel [27]. The deteriorating situation in the Gaza Strip in more recent years, with further reductions in the availability of essential medicines adding to long-term lack of services and facilities for particular patient groups, has significant potential implications for the morbidity and mortality of cancer patients. Further work is required to supplement current data with dates of diagnosis and staging/grading of tumours to produce internationally comparable cancer survival estimates. Moreover, while this study has shown the significant overall impact of permit delays and denials on patient mortality, additional studies are required to ascertain the differential impacts of such barriers to access on different cancer types.

In conclusion, this study highlights the critical importance of timely access for cancer patients to lifesaving chemotherapy and radiotherapy treatments and the need to minimize administrative processes that might delay such access. The permits system described in this study continues to apply for Palestinian patients in the Gaza Strip, as well as for most Palestinian patients from the West Bank outside of east Jerusalem. The majority of unsuccessful permit applications remain the result of delays in processing, with patients missing hospital appointments due to lack of any definitive response to their applications rather than denial for alleged security reasons. This study demonstrates the potential for use of existing administrative datasets to carry out epidemiological studies to assess the health impact of policies on Palestinian patients in Gaza. The findings of this research have implications for protection of the right to the highest attainable standard of health of Palestinians in the oPt, and reaffirm the need for stronger measures to safeguard access to essential health services for referral patients in the Gaza Strip [28, 29].

## Supporting information

S1 Table Data quality indicators for the total dataset from which cancer patient applications for chemotherapy and/or radiotherapy were extracted. (DOCX)

S1 Fig Timeline for Gaza patient referrals: 2008–2017. (PDF)

S2 Fig (JPG)
