# Supplementary material for: Comparative survival of cancer patients requiring Israeli permits to exit the Gaza Strip for health care: A retrospective cohort study from 2008 to 2017
Source: PLoS One. 2021 Jun 2;16(6):e0251058. doi: 10.1371/journal.pone.0251058 (PMC8172025; doi:10.1371/journal.pone.0251058)
Supplement: S1 Table — (DOCX) [file pone.0251058.s001.docx]

**S1 Table: Data quality indicators for the total dataset from which cancer patient applications for chemotherapy and/or radiotherapy were extracted**

| **Data quality indicator** | **Number of entries affected** | **Denominator** | **Percentage of entries affected** |
| --- | --- | --- | --- |
| Missing data on sex | 198 | Total number of patients (44,812) | 0.4% |
| Not present in population registry as being dead or alive | 59 | Total number of patients (44,812) | 0.1% |
| Missing data on diagnosis | 297 | Total number of applications (153,037) | 0.2% |
| Died before an application date | 37 | Total number of deaths (8,916) | 0.4% |
| Died more than 30 days before an application date | 11 | Total number of deaths (8,916) | 0.1% |
