# Supplementary material for: Comparative survival of cancer patients requiring Israeli permits to exit the Gaza Strip for health care: A retrospective cohort study from 2008 to 2017
Source: PLoS One. 2021 Jun 2;16(6):e0251058. doi: 10.1371/journal.pone.0251058 (PMC8172025; doi:10.1371/journal.pone.0251058)
Supplement: S1 Fig — (PDF) [file pone.0251058.s002.pdf]

TIMELINE FOR GAZA PATIENT REFERRALS: 2008-2017

World Health Organization
occupied Palestinian territory

Palestinian Ministry of Health (MoH) referrals

3,816 patients

17,072 permit applications to exit Gaza Strip

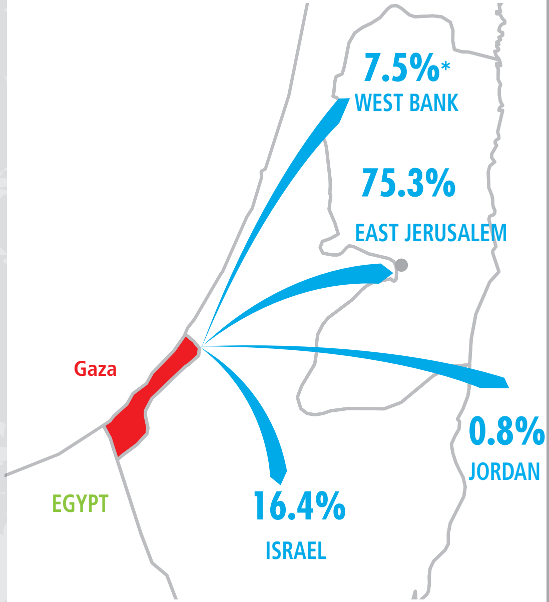

	**1 MEDICAL DECISION**	**2 MEDICAL APPROVAL**	**3 FINANCIAL APPROVAL**	**4 APPOINTMENT REQUEST**
	Doctor refers patient for treatment not available in Gaza MoH hospitals.	The Services Purchasing Unit (SPU) medically approves decision for referral.	SPU financially approves decision for referral.	SPU requests appointment from the receiving hospital and informs patient of their appointment
Regular cases	2-7 days	1-7 days	1-7 days	14-42 days
Urgent cases	1 day	1 day	1 day	2-3 days

5 PERMIT APPLICATION

2-5 hours

2-5 hours

Patient or their relatives request permit through Palestinian Health Liaison Office (HLO), which submits permit application to The Israeli Coordination and Liaison Administration to the Gaza Strip (CLA)

Israeli Permits:

83%

3,161 patients had thier initial application approved. 17% (655 patients) had thier application delayed or denied

59%

(2,254 of 3,816) patients who required permits were women

9.4% (360 of 3,816) patients who required permits were children 0-17 years old

6 PERMIT PROCESS

1 days

7-30 days

CLA processes permit applications. Patient waits for a response by text through the HLO, which usually comes a day before their appointment date.

7 CROSSING EREZ CHECKPOINT

5-7 hours

5-7 hours

Patient passes through the de facto authority checkpoint, Palestinian Authority registration post and Israeli (Erez) checkpoint to exit Gaza, including a 1km corridor from the Palestinian to the Israeli terminals. At the Erez terminal, patients undergo a body search, luggage search and permit checks. Patients and companions may be interrogated, arrested or detained.

8 RECEIVING CARE

90% of Gaza patients attending for healthcare require admission for in-patient stay. Further delays for patients can arise where services are overstretched and hospitals lack vacant beds.

9 TRAVEL BACK TO GAZA

5-7 hours

5-7 hours

Patients visiting West Bank hospitals must cross checkpoints into Jerusalem, as well as having to pass through Erez checkpoint into Gaza. Each checkpoint means further delays and the possibility of interrogation or arrest.

* West Bank here refers to the West Bank outside East Jerusalem.

The percentages in the figure are the destinations for first permit applications by patients referred for chemotherapy and/or radiotherapy between 1 January 2008 and 31 December 2017 (N=3,816)
