# Supplementary figures and images for: Comparative survival of cancer patients requiring Israeli permits to exit the Gaza Strip for health care: A retrospective cohort study from 2008 to 2017
Source: PLoS One. 2021 Jun 2;16(6):e0251058. doi: 10.1371/journal.pone.0251058 (PMC8172025; doi:10.1371/journal.pone.0251058)

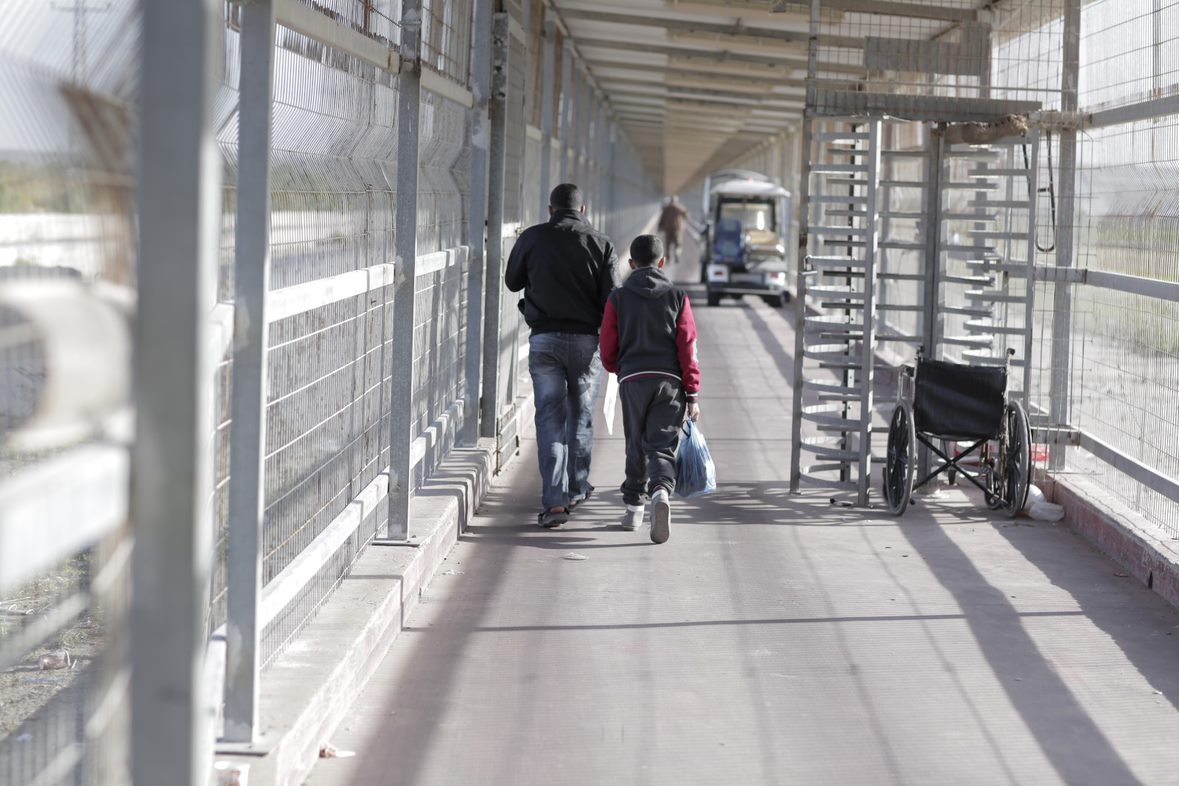

Supplement: S2 Fig — (JPG) [file pone.0251058.s003.jpg]
